# Choosing surgery: patients' preferences within a trial of treatments for anterior cruciate ligament injury. A qualitative study

**DOI:** 10.1186/1471-2474-10-100

**Published:** 2009-08-10

**Authors:** Carina A Thorstensson, L Stefan Lohmander, Richard B Frobell, Ewa M Roos, Rachael Gooberman-Hill

**Affiliations:** 1 Department of Research and Development, Spenshult Hospital for Rheumatic Diseases, Oskarström, Sweden; 2 Department of Orthopaedics, Clinical Sciences Lund, Lund University, Lund, Sweden; 3 Institute of Sports Science and Clinical Biomechanics, Faculty of Health Sciences, University of Southern Denmark, Odense, Denmark; 4 Clinical Science at North Bristol, Bristol University, Bristol, UK

## Abstract

**Background:**

The objective was to understand patients' views of treatment after acute anterior cruciate ligament (ACL) injury, and their reasons for deciding to request surgery despite consenting to participate in a randomised controlled trial (to 'cross-over').

**Methods:**

Thirty-four in-depth qualitative interviews were conducted with young (aged 18–35), physically active individuals with ACL rupture who were participating in a RCT comparing training and surgical reconstruction with training only. 22/34 were randomised to training only but crossed over to surgery. Of these, 11 were interviewed before surgery, and 11 were interviewed at least 6 months after surgery. To provide additional information, 12 patients were interviewed before randomisation. Interviews were audio-recorded, transcribed and analysed using the Framework approach.

**Results:**

Strong preference for surgery was commonplace and many patients said that they joined the RCT in order to bypass waiting lists. Patients who chose to cross-over described training as time consuming, boring and as unable to provide sufficient results within a reasonable timeframe. Some said their injured knees had given-way; others experienced new knee traumas; and many described their lack of trust in their knee. Patients believed that surgery would provide joint stability. Despite the ostensible satisfaction with surgery, more detailed exploration showed mixed views.

**Conclusion:**

Participants in a trial of treatments for acute ACL injury express a variety of views and beliefs about those treatments, and trial participation happens in the absence of equipoise. Furthermore, opting for surgical reconstruction does not necessarily provide patients with satisfactory outcomes. Definition of successful outcome may require an individualised approach, incorporating patients' as well as surgeons' views before treatment decisions are made.

## Background

Knee injury, including ACL injury, is a known risk factor for the development of knee osteoarthritis (OA), and it was estimated that about 50% of all individuals with an acute ACL injury develop knee OA within 10–15 years [1-4]. There is no consistent evidence to suggest that ACL reconstruction actually prevents the development of OA [1,5]. The lack of clear evidence about long-term consequences related to different treatments can partly be explained by factors precluding randomised controlled trials (RCTs) comparing surgical and non-surgical treatments, and the difficulties of recruitment into such trials. A lack of clinical equipoise, i.e. true uncertainty about which treatment is most effective, is common among surgeons and sometimes precludes surgical RCTs [6,7]. The influence of patients' preferences and perceptions of equipoise on compliance and willingness to participate is not well explored, and often based on hypothetical trials [8-10]. Even among participants who agree and understand both clinical equipoise and the process of randomisation, about 10–15% still have a preference for a particular treatment and hope to be randomised to that particular treatment arm [8,11].

Trial participants' decisions to leave their allocated intervention arm and 'cross-over' to an alternative intervention confounds interpretation of outcome and has been described as a possible problem in trials of surgical treatments [7]. In a trial comparing surgical and non-surgical intervention for lumbar disc herniation, 30% of participants in the non-surgical treatment arm crossed over to the surgical treatment arm [12]. Studies exploring in-depth the reasons for cross-over in musculoskeletal trials are rare. Our study aimed to understand patients' views about treatment after acute ACL injury, and to explore why patients crossed over from the training only to the surgical and training treatment arm despite consenting to participate in a trial comparing the two treatments.

## Methods

Participants in this qualitative study were recruited from an ongoing RCT: the KANON-study (ISRCTN 84752559, http://www.controlled-trials.com). The RCT aims to explore short term (2 years) and long term (5 years) effects of surgical reconstruction plus training or training only. Patients aged 18 to 35 years, with a moderate to high level of physical activity and with a complete rupture of the ACL were randomised either to arthroscopic surgical reconstruction followed by physiotherapist supervised outpatient training (exercise), or to supervised training only. Physical activity was assessed using the Tegner activity rating scale [13], graded from 1 (activities like walking on even ground, playing cards) to 10 (football, rugby, wrestling at a high (national) level of competition). Moderate to high level of physical activity was defined as 5–9 on the Tegner activity rating scale. The presence of ACL injury was confirmed with magnetic resonance imaging (MRI), and patients were recruited to the RCT within four weeks of their injury. A complete description of inclusion and exclusion criteria is provided elsewhere [14]. The ACL reconstruction was performed within 6 weeks after randomisation using bone-patella tendon-bone (BTB) autografts [15] or quadruple hamstrings (Semitendinosus/Gracilis tendon) autografts [16]. The training protocol was based on a consensus report of rehabilitation of ACL injured subjects, developed by the Swedish Association of Sports Medicine and consistent with published literature [17], and identical for all subjects regardless of treatment arm. The training was moderately aggressive and supervised by physical therapists. Pain, swelling and general discomfort slowed down the progression. Those treated with ACL reconstruction therefore proceeded at a slower pace through the first 2–3 months after surgery. Before randomisation all participants received a DVD containing an interview with an ACL-injured patient not included in the RCT, and a panel discussion with three experienced orthopedic surgeons regarding scientific evidence and clinical opinions for and against the two treatment options [14].

In the clinical trial 19% of potential participants declined to take part due to an existing preference for one of the treatments [14]. The number who declined participation because they were unwilling to be randomised to surgery (n = 23) was 3-fold the number of patients unwilling to be randomised to the training-only arm (n = 8).

### Participants

**Figure 1:**
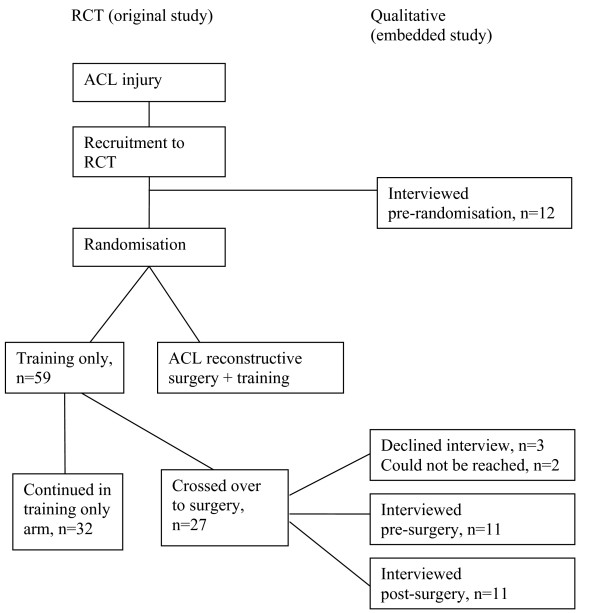
**Flowchart of recruitment to qualitative interviews**.

As this qualitative study aimed to explore the issue of cross-over, participants comprised those individuals who had been randomised to the non-surgical treatment (training only) arm but who had subsequently decided to have surgery. The Knee Injury and Osteoarthritis Outcome Score (KOOS) [18,19] was completed at the time point for cross-over to assess participants' knee symptoms. December 31^st ^2007 was regarded as "end-point" for inclusion in the present study. Of the 59 trial participants randomised to the training only arm, 27 (46%) had crossed-over to surgical reconstruction by the end of 2007 (figure 1). All 27 were invited to take part in this study, some of them before their surgery, and some of them afterwards. On average, cross-over was taking place 14 months (range 3–52) after inclusion (table 1). In addition, a group of patients who had consented to take part in the trial but who had not yet been randomised were approached (figure 1). This provided additional information about the views of patients at a time when their opinions were as yet unaffected by the experience of participation in either treatment arm. Patients gave written informed consent to take part, and ethical approval was provided by Lund University (LU 535-01).

**Table 1:** Characteristics of participants in qualitative interviews

ID	Sex	Age	Self-rated pre-injury Tegner activity score	Months in training before cross-over	Troubled by lack of confidence in knee at cross-over*
Pre-surgery group					
KA1013	F	25	9	52	Severely
KA1081	F	27	9	27	Extremely
KA1155	M	23	9	11	Severely
KA2105	M	32	7	8	Extremely
KA2111	F	20	6	12	Moderately
KC1042	M	34	8	41	Severely
KC1046	F	28	8	10	Severely
KC1050	F	25	9	20	Severely
KC1121	M	29	8	8	Severely
KC2066	M	28	7	6	Severely
KC2068	M	24	7	11	Moderate
Post-surgery group					
KA1001	M	29	8	3	Mildly
KA1002	M	37	8	16	Severely
KA1007	F	26	8	7	Moderately
KA1008	M	29	9	13	Moderately
KA1018	M	30	8	25	Extremely
KA1083	F	35	8	5	Mildly
KA1147	M	28	9	6	Extremely
KA1153	M	27	9	6	Severely
KA2027	M	38	7	6	Moderately
KA2031	M	26	7	11	Severely
KC1043	M	24	9	6	Extremely
Pre-randomisation group					
KA1166	M	24	8		
KA1167	M	23	9		
KA1168	M	21	9		
KA1169	F	27	9		
KA1170	M	31	9		
KA2103	F	34	7		
KA2105	M	32	8		
KC1122	M	35	9		
KC1123	M	22	9		
KC2066	M	28	6		
KC2067	M	25	7		
KC2075	F	22	7		

*Self-rated, item from Knee injury and Osteoarthritis Outcome Score [18,19], possible answers: not at all, mildly, moderately, severely, extremely

#### Pre-surgery cross-over group

All patients in the training only treatment arm who opted for surgery between January 2006 and December 2007 (n = 12) were contacted by telephone and invited to the pre-surgery group. Eleven patients (6 men and 5 women) consented to take part and only one (a woman) declined participation. All except one of the patients in this group were interviewed prior to surgery. The participant (KA1081) who was interviewed after surgery had undergone surgery just two days prior to interview and the topics covered in her interview were the same as those covered with the rest of the group. Interviews with this group focused on experiences of the training intervention and reasons for requesting surgery.

#### Post-surgery cross-over group

Patients in the training only group who opted for surgery and who had an ACL reconstruction at least 6 months prior to the start of the qualitative study (n = 15) were invited to take part in the post-surgery group. Eleven patients (9 men and 2 women) agreed to be interviewed. Two could not be reached by telephone (one man and one woman). One man preferred not to be interviewed, and one man did not attend the interview and thereafter could not be reached. Participants in this group were interviewed, between 6 months and 2 years after having surgery, about their reasons for requesting for surgery, and their views and experience of treatment of ACL injury.

#### The pre-randomisation group

The final fourteen patients recruited to the RCT were contacted by telephone before randomisation and were interviewed about their experiences of ACL injury and treatment preferences. Two interviews were excluded from analysis: one due to recording failure, and one woman who had already been randomised at the time of interview. This left 12 patients (9 men, 3 women) in the pre-randomisation group.

Two of the men (KC2066, KA2105) interviewed in the pre-randomisation group were randomised to training only but opted for surgery during the study. They also agreed to be interviewed before their surgery as well. In total, 34 interviews were conducted with 32 people, 22 men and 10 women, aged 20–38 (Table 1).

Interviews with members of the post-surgery group were performed face-to-face at a place chosen by the participant. Because of limited time between a participant's decision to cross-over and their date for surgery in the pre-surgery group, and from a participant's agreement to take part in the RCT and their allocation to a treatment arm in the pre-randomisation group, interviews had to be scheduled and conducted with some speed while also at the convenience of the participants. Interviews with members of the pre-surgery and the pre-randomisation groups were therefore conducted over the telephone.

Each interview lasted between 30 and 60 minutes and was performed by a physical therapist (CT) with training in qualitative research methods and who was not otherwise involved in the trial. An interview topic guide with open-ended questions was used for all interviews. Questions included: *Tell me about your injury? How should ACL injuries be treated? Why did you join the randomised controlled trial*? For the pre- and post-surgery groups the following questions were added: *What do/did you think of your treatment? Why do/did you want surgery*? *What do you think about your future participation in sports*? Themes brought up by the participants were followed-up by probing. All interviews were audio-recorded, and conducted and transcribed verbatim in Swedish. Transcripts were translated into English by the interviewer (CT).

### Analysis

Data was analysed using the Framework approach [20,21]. Briefly, the analysis began with reading and re-reading the transcripts and listening to the audio-recordings to obtain an overview of the data. The data was then coded inductively (indexing), and an index list was developed. The index codes were then further refined and applied systematically to all data. Content-related index codes were grouped together into categories, which were revised as the analysis progressed. Subsequently, the data was organised into "charts", or tables, to allow comparison within and between cases and categories. During this stage the original context was revisited several times to ensure appropriate assignment of data. Patterns were identified, further refined to describe the conceptions of the participants, and illustrated with quotations from the interviews. To ensure the appropriate assignment of indexed data, transcripts and charts were cross-checked by an experienced qualitative researcher (RG-H) on multiple occasions.

## Results

Participants in the pre-surgery and post-surgery groups described reasons for their decision to opt for surgery despite having been randomised to the non-surgical intervention arm. These reasons were grouped into three main categories: *a strong pre-existing preference for surgery; a poor experience/outcome of training; a desire to regain pre-injury status*. By comparison, participants in the pre-randomisation group discussed the preference for surgery and their desire to regain pre-injury status and possible determinants for outcome of training. In all three groups surgery was seen as facilitating return to sporting activity, although participants also discussed the possibility of surgery failing and the future risk of developing OA. A common view among members of all three groups was the belief that their own recovery time would be shorter than average.

### A strong pre-existing preference for surgery

Participants in all three groups were more aware of surgery and training than training only as a form of treatment for ACL injury. Surgery was described as necessary to achieve joint stability and thereby to prevent re-occurrence of injury:

I think ACL injuries should have surgical treatment. I'm 25 and have my whole life to live. If I don't have surgery there might be things I can't do in future (KC1050, female, pre-surgery group)

Some of those who opted for surgery described regret that they had not had it sooner:

If I had had surgery from the beginning I would probably not have had the new injury of the meniscus and ligament, and maybe the outcome of surgery would have been better (KA1013, female, pre-surgery group)

Although surgery was seen as desirable, participants were aware of the possibility of failure and of future risk of developing OA:

I imagine the risk of OA increases with surgery. It might be worth the risk if you think you'll have an additional 10 years with football, because it's a long period of your life. If you have OA later, you might be prepared to deal with it, because you had 10 more years with football (KA1001, male, post-surgery group)

Several participants in the pre- and post-surgery groups said that they had wanted surgery from the beginning. This was particularly clear when members of the post-surgery group reflected on their reasons for choosing surgery. Some felt persuaded to take part in the RCT:

I wanted surgery from the beginning, but I felt persuaded to try training after randomisation. I had surgery in the back of my head all the time, even when I did give training a decent go (KC1043, male, post-surgery group)

Others joined the study with a view to bypassing waiting lists and obtaining access to particularly skilled surgeons:

I saw the study as a faster way to surgery, no matter what treatment arm I was randomised into (KA1083, female, post-surgery group)

In the pre-randomisation group, participants were split between those who expressed a preference for surgery, those who said that they would prefer training and those who said that they did not prefer one treatment over the other. Among those who described a preference for surgery, some used a metaphor of 'repair':

I would like to have surgery done, then it feels as if you've done what you could, seen what's broken and had it repaired (KC2066, male, pre-randomisation and pre-surgery groups)

Preference for training only treatment was sometimes based on concern about surgery:

I am a bit sceptical about having them cut my knee, or whatever they do when they do this surgery. It's better to have it done more naturally. [I] don't like them to rummage about in my knee, [I] think it might feel worse afterwards (KC1122 male, pre-randomisation group)

Those who did not express a preference for one treatment over the other talked about the uncertainty about the value of one treatment over the other and described 'trust' in the doctors:

I don't care, since they don't know, but I trust the doctors (KC2075, female, pre-randomisation group)

Participants were pragmatic in their approach to treatment, describing a desire to try the non-surgical option first since they could still opt for surgery if training was not successful, and then they had tried all treatments available. Participants in the pre- and post-surgery groups all expressed this view as did participants who were still awaiting randomisation:

You can't undo surgery, but if you have training and don't get well you can always have surgery (KC2075, female, pre-randomisation group)

### A poor experience/outcome of training

Participants who had undergone the training intervention but who had then opted for surgery discussed their experience of training. Several described training as boring or as too time consuming, both of which presented obstacles:

I don't want to keep doing exercises to have a knee that is OK. It's not fun. I want to be able to do what I want without thinking about what I do (KA2111, female, pre-surgery group)

The supervised training was mainly daytime, and I have been working a lot and can't find the time. Then I had my second child, and there are just not enough hours (KC1042, male, pre-surgery group)

Some did well after non-surgical treatment but felt that they would have needed to continue exercising if they wanted to see more improvement:

I think I might have needed another couple of months to build up more strength. I still think I could have made it without [an] ACL (KA1018, female, post-surgery group)

Others talked about training helping them cope but not enabling them to reach their full potential:

*Training was good, it helped me cope at home, to get stronger and dare more. But I reached the line where I felt good but not 100%. I have been somewhat better, but I want to achieve the last bit. It may get worse as well, they've told me that, even though the chances are quite good. 90–95% get well from surgery, that's quite a lot (KC2068, male, pre-surgery group)*.

For some the lack of improvement with training had an impact on mood:

I did an honest attempt to do training 3 days per week for a couple of months, without improvement. I thought about getting back to work 5 months after injury, but after 3 months I knew that was not going to happen. The last 2 months nothing happened. I felt hopeless and got into a depression (KC1043, male, post-surgery group)

Outcomes of treatment were sometimes viewed as partly beyond participants' control. In these instances, participants described results as related to external factors like anatomical differences, type of injury, surgeons' skills, luck, and healing ability:

If I fail, it might have something to do with my ability to heal, not with my training.... My muscles might be too weak or not be able to grow as fast as it takes (KA1167, male, pre-randomisation group)

For others, the occurrence of knee trauma influenced the outcome:

I was very pleased with the results from training until I slipped on ice. My legs disappeared, and I tried to keep the balance. It was bad luck, I got all my weight on my injured knee, and it gave way. (KC1046, female, pre-surgery group)

Other participants were convinced that their effort, self-discipline, and whether they followed the regime were crucial to success:

*I had 6 months of supervised training, and then I went abroad and was supposed to do exercises on my own. Maybe if I had had a couple of more months to increase the strength sideways and in [collateral] ligaments. It's my fault; I was the one who moved away (KA2111, female, pre-surgery group)*.

A common belief among participants in all groups was that their own recovery time would be shorter than average, because of their motivation to succeed and their pre-existing good physical condition:

I've always built muscles very easy, so I might not need 6 months of rehab (KC2075, female, pre-randomisation group)

### A desire to regain pre-injury status

Participants from all three groups described their desire to continue with sporting activities. In the pre- and post-surgery groups participants also expressed dissatisfaction with sporting ability following the training intervention. Concerns included inability to perform sports at previous levels, and the experience of their injured knee 'giving way' during activities. Along with existing beliefs about the value of surgery, these concerns contributed to the decision to opt for surgery:

I want to be able to do things like football just for fun without fear of the knee giving way. I don't want to be worried about future injuries either. I had multiple injuries, and I think the odds of succeeding with training were bad because of that. (KA2105, male, pre-surgery group)

In describing failure to perform sports, participants described their efforts to return to previous activity levels:

It felt good after training, but it didn't last through the real test [football] (KA2027, male, post-surgery group)

When I started to play football I noticed it wasn't as good as I first thought. I could run without problems, but I didn't feel stable playing football. I thought it would get better, but even if I trained hard it didn't work, so I wanted surgery (KA1008, male, post-surgery group)

Episodes of the injured knee 'giving way' during sports was said to be the precursor for ongoing stability problems:

I felt good, but then I started to play [indoor bandy] again. I played a game, and it went really well almost the whole game, but towards the end I had an unlucky tackle. I had a give-way, and since then the instability has been a lot worse (KC1121, male, pre-surgery group)

Not everyone described an episode of give-way, but many talked about lack of stability and lack of trust in the ability of their injured knee to provide support particularly on 'uneven' ground:

The knee felt unstable despite all the training. It was unstable when I cut the lawn and walked on uneven ground. (KA2031, male, post-surgery group)

I could run and play some football, but not walk in the woods (KA1083, female, post-surgery group)

The desire to do spontaneous activities in future without thinking about their knee was seen as an important reason for having surgery:

I want to be able to play for fun when I get older as well (KA1018, male, post-surgery group)

Participants who were interviewed following surgery described their views on their outcome. Four of the eleven participants interviewed 6 months or more after surgery said they were satisfied with the results, while 7 were somewhat dissatisfied. However, these statements appear to mask more mixed views about outcome, for instance, two of the participants who said they were satisfied never returned to their previous sporting activities. Dissatisfaction was related to pain, lack of sensation, difficulties kneeling, swelling after activities, and crepitus. Participants adapted or revaluated their lifestyle to cope with the results.

## Discussion

Patient preferences are known to affect recruitment to randomised trials. Patients who prefer one of the possible treatments to the other(s) are less likely to consent to take part [8,11]. Lack of patient equipoise has been shown in other randomised controlled trials comparing surgical and non-surgical interventions [7,12]. This qualitative study shows that those participants who agreed to be randomised but who had strong preferences for surgery saw trial participation as a means to circumventing waiting lists. However, this is possibly a phenomenon applicable only to countries where health care is incorporated in the welfare system, and high frequencies of scheduled surgeries result in waiting lists and delayed surgery. On the other hand, waiting lists in standard care could, if combined with training, allow for patients to try rehabilitation before decisions about surgical treatment are made. A screening examination might also be a helpful tool to in the decision-making to determine the chances for physically active individuals to succeed with non-surgical treatment [22,23]. Some participants in this study decided to 'risk' randomization to the training alone intervention as a first line of approach, but felt that surgery remained an option for them if training did not confer the desired results in an acceptable timeframe. A recent prospective study that aimed to identify potential "copers", i.e. patients who after a screening examination were considered as likely to succeed with non-surgical treatment, showed that 40% of those who were classified as copers still opted for surgical reconstruction [23]. This might be explicable by our findings which indicate that the reasons behind patients' decisions to opt for surgery may in fact relate to strategy and pre-existing preferences rather than ability to cope.

There is a strong preference for ACL reconstructive surgery within the orthopaedic surgeon community in the US, in Canada, and in Europe [6,24,25]. It is a common belief that an ACL reconstruction is needed to participate in sports, also on lower recreational levels, although evidence from high quality scientific trials in support of such beliefs are lacking [5]. Current sports medicine literature recommends return to pre-injury sports activities approximately 6 months after ACL surgery [26]. Despite ostensible satisfaction with surgery in this study, more detailed exploration of experience showed patients' expectations were often not met. This may be related to the belief that surgery could provide pre-injury status alongside belief in personal ability to recover more quickly than others. The delayed recovery and return to sports after ACL reconstruction surgery are known to cause frustration [27]. Our results show that some patients also feel this way about training treatment alone, as several participants thought it was possible for them to recover faster than average. Failing to fulfil these expectations caused disappointment and frustration, reduced confidence in training and reinforced the desire for surgery. This is supported by the findings from a recently published study showing that 72% of patients who opted for and underwent non-surgical ACL treatment successfully returned to high-level sport activities in the short term [23]. However, 10 years later 67% had undergone surgical reconstruction.

Return to pre-injury level of sports is often used as an outcome measure of successful treatment after ACL injury [28-31], and patients often believe that surgical reconstruction of the ACL is needed to be able to practice sports [27]. The return to sport after ACL injury is a much debated outcome [32]. In the present study, which included physically active people, everyone expressed a desire to return to sports. Stability was seen as crucial for successful return to sports, and therefore was an important treatment goal. Although stability alone does not explain the return to sports [33] and people's views about the importance of return to sports shift during the time they are injured [27], patients' views about how they may achieve optimal outcomes are key to treatment decisions.

The in-depth qualitative interviews enabled participants to describe and define their own experience of ACL injury, treatment, trial participation and cross-over. Furthermore, by the end of data collection no new themes were emerging from the data, which indicates that 34 interviews was an adequate number for this study. By selecting patients from three groups (pre-randomisation, pre- and post-surgery) we were able to explore views from participants at different stages in the clinical trial process. Since the study aimed to examine cross-over, we did not include trial participants who had been randomised and had remained in their allocated arm, whereas inclusion of participants prior to randomisation provided valuable data about preferences regarding treatment options that was still unaffected by the experience of one treatment arm or another. Future work might aim to follow trial participants from both training and surgical arms through the process of a trial in a longitudinal, prospective design.

## Conclusion

This study shows that participants in a trial of treatments for acute ACL injury express a variety of views and beliefs about those treatments, and trial participation happens in the absence of equipoise. Furthermore, opting for surgery does not necessarily provide patients with satisfactory outcomes, and this may be related to expectations about stability, return to sports and ability to recover. This suggests that definition of successful outcome may require an individualised approach, incorporating patients' as well as surgeons' views before treatment decisions are made.

## Competing interests

The authors declare that they have no competing interests.

## Authors' contributions

CT participated in study design, data collection, analysis and writing of the manuscript. SL, ER and RF participated in design, interpretation of data, and critical revision of the manuscript. RG-H participated in design, analysis, interpretation of data, and critical revision of the manuscript. All authors read and approved the final manuscript.

## Pre-publication history

The pre-publication history for this paper can be accessed here:

http://www.biomedcentral.com/1471-2474/10/100/prepub

## References

[B1] Lohmander LS Englund PM Dahl LL Roos EM The long-term consequence of anterior cruciate ligament and meniscus injuries: osteoarthritis Am J Sports Med 2007 10 1756 69 10.1177/0363546507307396 17761605

[B2] Englund M Roos EM Lohmander LS Impact of type of meniscal tear on radiographic and symptomatic knee osteoarthritis: a sixteen-year followup of meniscectomy with matched controls Arthritis Rheum 2003 10 2178 87 10.1002/art.11088 12905471

[B3] Roos EM Joint injury causes knee osteoarthritis in young adults Curr Opin Rheumatol 2005 10 195 200 10.1097/01.bor.0000151406.64393.00 15711235

[B4] Roos H Adalberth T Dahlberg L Lohmander LS Osteoarthritis of the knee after injury to the anterior cruciate ligament or meniscus: the influence of time and age Osteoarthritis Cartilage 1995 10 261 7 10.1016/S1063-4584(05)80017-2 8689461

[B5] Linko E Harilainen A Malmivaara A Seitsalo S Surgical versus conservative interventions for anterior cruciate ligament ruptures in adults Cochrane Database Syst Rev 2005 CD001356 15846618 10.1002/14651858.CD001356.pub3

[B6] Marx RG Jones EC Angel M Wickiewicz TL Warren RF Beliefs and attitudes of members of the American Academy of Orthopaedic Surgeons regarding the treatment of anterior cruciate ligament injury Arthroscopy 2003 10 762 70 12966385 10.1016/s0749-8063(03)00398-0

[B7] McCulloch P Taylor I Sasako M Lovett B Griffin D Randomised trials in surgery: problems and possible solutions BMJ 2002 10 1448 51 10.1136/bmj.324.7351.1448 12065273 PMC1123389

[B8] Mills N Donovan JL Smith M Jacoby A Neal DE Hamdy FC Perceptions of equipoise are crucial to trial participation: a qualitative study of men in the ProtecT study Control Clin Trials 2003 10 272 82 10.1016/S0197-2456(03)00020-5 12757993

[B9] Robinson EJ Kerr CE Stevens AJ Lilford RJ Braunholtz DA Edwards SJ Beck SR Rowley MG Lay public's understanding of equipoise and randomisation in randomised controlled trials Health Technol Assess 2005 10 1 192 15763039 10.3310/hta9080

[B10] Ubel PA Merz JF Shea J Asch DA How preliminary data affect people's stated willingness to enter a hypothetical randomized controlled trial J Investig Med 1997 10 561 6 9444883

[B11] Creel AH Losina E Mandl LA Marx RJ Mahomed NN Martin SD Martin TL Millett PJ Fossel AH Katz JN An assessment of willingness to participate in a randomized trial of arthroscopic knee surgery in patients with osteoarthritis Contemp Clin Trials 2005 10 169 78 10.1016/j.cct.2004.12.010 15837439

[B12] Weinstein JN Tosteson TD Lurie JD Tosteson AN Hanscom B Skinner JS Abdu WA Hilibrand AS Boden SD Deyo RA Surgical vs nonoperative treatment for lumbar disk herniation: the Spine Patient Outcomes Research Trial (SPORT): a randomized trial JAMA 2006 10 2441 50 10.1001/jama.296.20.2441 17119140 PMC2553805

[B13] Tegner Y Lysholm J Rating systems in the evaluation of knee ligament injuries Clin Orthop Relat Res 1985 10 43 9 4028566

[B14] Frobell RB Lohmander LS Roos EM The challenge of recruiting patients with anterior cruciate ligament injury of the knee into a randomized clinical trial comparing surgical and non-surgical treatment Contemp Clin Trials 2007 10 295 302 10.1016/j.cct.2006.10.002 17137844

[B15] Jones KG Reconstruction of the anterior cruciate ligament using the central one-third of the patellar ligament J Bone Joint Surg Am 1970 10 838 9 5479469

[B16] Aglietti P Buzzi R Menchetti PM Giron F Arthroscopically assisted semitendinosus and gracilis tendon graft in reconstruction for acute anterior cruciate ligament injuries in athletes Am J Sports Med 1996 10 726 31 10.1177/036354659602400605 8947392

[B17] Holm I Fosdahl MA Friis A Risberg MA Myklebust G Steen H Effect of neuromuscular training on proprioception, balance, muscle strength, and lower limb function in female team handball players Clin J Sport Med 2004 10 88 94 10.1097/00042752-200403000-00006 15014342

[B18] Roos EM Roos HP Ekdahl C Lohmander LS Knee injury and Osteoarthritis Outcome Score (KOOS) – validation of a Swedish version Scand J Med Sci Sports 1998 10 439 48 9863983 10.1111/j.1600-0838.1998.tb00465.x

[B19] Roos EM Roos HP Lohmander LS Ekdahl C Beynnon BD Knee Injury and Osteoarthritis Outcome Score (KOOS) – development of a self-administered outcome measure J Orthop Sports Phys Ther 1998 10 88 96 9699158 10.2519/jospt.1998.28.2.88

[B20] Ritchie J Spencer L Bryman A, Burgess R Qualitative data analysis for applied policy research Analyzing Qualitative Data 1994 London: Routledge

[B21] Ritchie J Lewis J Qualitative Research Practice: a guide for social science students and researchers 2003 London: Sage

[B22] Fitzgerald GK Axe MJ Snyder-Mackler L A decision-making scheme for returning patients to high-level activity with nonoperative treatment after anterior cruciate ligament rupture Knee Surg Sports Traumatol Arthrosc 2000 10 76 82 10.1007/s001670050190 10795668

[B23] Hurd WJ Axe MJ Snyder-Mackler L A 10-year prospective trial of a patient management algorithm and screening examination for highly active individuals with anterior cruciate ligament injury: Part 1, outcomes Am J Sports Med 2008 10 40 7 10.1177/0363546507308190 17940141 PMC2891099

[B24] Mirza F Mai DD Kirkley A Fowler PJ Amendola A Management of injuries to the anterior cruciate ligament: results of a survey of orthopaedic surgeons in Canada Clin J Sport Med 2000 10 85 8 10.1097/00042752-200004000-00001 10798788

[B25] Kapoor B Clement DJ Kirkley A Maffulli N Current practice in the management of anterior cruciate ligament injuries in the United Kingdom Br J Sports Med 2004 10 542 4 10.1136/bjsm.2002.002568 15388535 PMC1724936

[B26] Kvist J Rehabilitation following anterior cruciate ligament injury: current recommendations for sports participation Sports Med 2004 10 269 80 10.2165/00007256-200434040-00006 15049718

[B27] Heijne A Axelsson K Werner S Biguet G Rehabilitation and recovery after anterior cruciate ligament reconstruction: patients' experiences Scand J Med Sci Sports 2007 10 325 35 18067526 10.1111/j.1600-0838.2007.00700.x

[B28] Kvist J Ek A Sporrstedt K Good L Fear of re-injury: a hindrance for returning to sports after anterior cruciate ligament reconstruction Knee Surg Sports Traumatol Arthrosc 2005 10 393 7 10.1007/s00167-004-0591-8 15703963

[B29] Barrett GR Noojin FK Hartzog CW Nash CR Reconstruction of the anterior cruciate ligament in females: A comparison of hamstring versus patellar tendon autograft Arthroscopy 2002 10 46 54 11774141 10.1053/jars.2002.25974

[B30] Meunier A Odensten M Good L Long-term results after primary repair or non-surgical treatment of anterior cruciate ligament rupture: a randomized study with a 15-year follow-up Scand J Med Sci Sports 2007 10 230 7 17501866 10.1111/j.1600-0838.2006.00547.x

[B31] Thomee P Wahrborg P Borjesson M Thomee R Eriksson BI Karlsson J Self-efficacy, symptoms and physical activity in patients with an anterior cruciate ligament injury: a prospective study Scand J Med Sci Sports 2007 10 238 45 16774652 10.1111/j.1600-0838.2006.00557.x

[B32] Myklebust G Bahr R Return to play guidelines after anterior cruciate ligament surgery Br J Sports Med 2005 10 127 31 10.1136/bjsm.2004.010900 15728687 PMC1725142

[B33] Eastlack ME Axe MJ Snyder-Mackler L Laxity, instability, and functional outcome after ACL injury: copers versus noncopers Med Sci Sports Exerc 1999 10 210 5 10.1097/00005768-199902000-00002 10063808

